# Комплексный подход к лечению претибиальной микседемы на основе пульс-терапии преднизолоном и электронейромиостимуляции (FREMS-терапии) у пациентки с болезнью Грейвса и эндокринной офтальмопатией

**DOI:** 10.14341/probl12888

**Published:** 2023-08-30

**Authors:** М. А. Перепелова, Е. Л. Зайцева, Е. Г. Бессмертная, Я. О. Груша, Н. Ю. Свириденко, Г. Р. Галстян

**Affiliations:** Национальный медицинский исследовательский центр эндокринологии; Национальный медицинский исследовательский центр эндокринологии; Национальный медицинский исследовательский центр эндокринологии; Научно-исследовательский институт глазных болезней им. М.М. Краснова; Национальный медицинский исследовательский центр эндокринологии; Национальный медицинский исследовательский центр эндокринологии

**Keywords:** болезнь Грейвса, претибиальная микседема, эндокринная офтальмопатия, электронейромиостимуляция (FREMS-терапия)

## Abstract

Болезнь Грейвса (БГ) относится к актуальным проблемам современной эндокринологии, характеризуется высокой частотой, полисистемным поражением организма, неуклонно прогрессирующим течением, диагностическими сложностями, высокой степенью инвалидизации, нередко — резистентностью к терапии. К проявлениям заболевания относят: синдром тиреотоксикоза c нарушением обмена липидов, углеводов, активацией полиорганной патологии в виде эндокринной офтальмопатии (ЭОП), претибиальной микседемы, сердечно-сосудистой недостаточности, акропатии, поражений нервной, костно-суставной системы, а также другие нарушения. Развитие полиорганной патологии может иметь разную последовательность, разные временные промежутки и разную степень выраженности. Любые разработки в направлении уточнения этиопатогенетических, клинико-диагностических и лечебно-реабилитационных мероприятий представляют несомненную значимость. Нами представлен клинический случай БГ, ЭОП и претибиальной микседемы, при котором был апробирован комплексный подход в тактике лечения претибиальной микседемы (комбинация пульс-терапии преднизолоном и электронейромиостимуляции — FREMS-терапии), в результате которого были получены положительные результаты в течение короткого времени.

## Abstract (en)

Graves’ disease (GD) is one of the urgent problems of modern endocrinology, characterized by a high frequency, polysystemic damage to the body, a steadily progressive course, diagnostic difficulties, a high degree of disability and often resistance to therapy. The manifestations of the disease include: thyrotoxicosis syndrome with impaired lipid and carbohydrate metabolism, and activation of multiple organ pathology in the form of thyroid eye disease (TED), pretibial myxedema, cardiovascular insufficiency, acropathy, lesions of the nervous, osteoarticular system, and other lesions. The development of multiple organ pathology can have a different sequence, different time intervals and different degrees of severity. Any developments in the direction of clarifying the etiopathogenetic, clinical diagnostic and treatment-rehabilitation measures are of undoubted significance. We present a clinical case of GD, TED and pretibial myxedema, in which an integrated approach was tested in the tactics of treating pretibial myxedema (a combination of pulse therapy with prednisolone and FREMS-therapy), as a result of which positive results were obtained within a short time.

## АКТУАЛЬНОСТЬ

Претибиальная микседема — редкое проявление аутоиммунной патологии щитовидной железы (ЩЖ) на фоне ее гипо- или гиперфункции, нередко сопряженное с эндокринной офтальмопатией (ЭОП) [1]. Патогенез заболевания до конца не изучен. Считается, что стимуляция фибробластов кожи цитокинами, продуцируемыми лимфоцитами, увеличивает объем матрикса в соединительной ткани, что приводит к сдавлению лимфатических сосудов и появлению отека. Коллагеновые тяжи в соединительной ткани распадаются и фрагментируются, что провоцирует лимфоцитарную инфильтрацию дермы. Муциновые инфильтраты, разделяющие коллагеновые волокна, и накопление кислых мукополисахаридов приводят к задержке воды и соли [2]. Длительное аутоиммунное воздействие может привести к тяжелому течению патологии с последующей инвалидизацией [3]. Частота развития патологии не превышает 4,5%, вследствие этого крупные рандомизированные исследования с оценкой различных методов лечения этого состояния отсутствуют. Ранее были описаны клинические случаи с описанием различной тактики ведения пациентов с претибиальной микседемой: терапия гиалуронидазой, глюкокортикоидами (ГК), тепротумумабом и т.д. [4].

В данной статье проведен анализ клинического случая с проявлениями стойкой и выраженной претибиальной микседемы, а также комплексного подхода к коррекции кожных нарушений на основе применения пульс-терапии преднизолоном в сочетании с FREMS-терапией (англ. frequency rhythmic electrical modulation system).

Данный метод применяется для лечения болевой формы дистальной полинейропатии, хронической венозной недостаточности, имеются данные о лечении трофических язв нижних конечностей, также описан клинический случай успешного применения при нейролипоматозе (болезни Беркума) [5]. FREMS-терапия включает генерирование биосовместимых электрических сигналов с помощью компьютеризированных нейростимуляторов и их воздействие через чрескожные электроды. Высокое отрицательное напряжение позволяет осуществлять деполяризацию клеточной мембраны и открывает ионные каналы, реактивируя физиологический ответ и стимулируя восстановление естественного гомеостаза клетки. За счет данных эффектов достигается усиление локального кровотока (в том числе за счет повышения экспрессии сосудистого эндотелиального фактора роста (VEGF)) [6], уменьшение межтканевого отека [7].

У данной пациентки FREMS-терапия была назначена эмпирически, на основании имеющихся данных об эффективности и безопасности при лечении трофических язв голени и других патологий нижних конечностей [8–10].

## ОПИСАНИЕ СЛУЧАЯ

Пациентка А., 66 лет, поступила в отдел терапевтической эндокринологии с жалобами на правосторонний экзофтальм, бинокулярную диплопию, ретробульбарные боли, покраснение и отек голеней.

Из анамнеза известно, что в 2006 г. выявлена БГ. Инициирована терапия тиреостатиками в различных дозировках, при попытке отмены препарата — рецидив. В 2011 г. выполнена субтотальная резекция ЩЖ, назначена заместительная терапия левотироксином натрия.

С 2016 г. стала отмечать ухудшение зрения, с ноября 2017 г. появились периорбитальные отеки, двоение. Проводились парабульбарные инъекции дексаметазона № 10, дипроспана № 4 (апрель-май 2018 г.), терапия пероральными ГК с незначительным положительным эффектом. При обследовании в марте 2018 г. проведено УЗИ ЩЖ — выявлена остаточная ткань обоих долей, справа — 10×8,5 мм, слева 8×11 мм. По данным лабораторных анализов от 15.03.2018 г.: свободный тироксин (св. Т4) — 15,2 пмоль/л (9–20), свободный трийодтиронин (св. Т3) — 4,1 пмоль/л (2,5–5,5), тиреотропный гормон (ТТГ) — 0,536 мЕд/л (0,4–4,0), антитела к рецептору ТТГ — 14,6 Ед/л (0–1.5), антитела к тиреопероксидазе (ТПО) — 17 Ед/мл (0–34) на фоне приема 88 мкг левотироксина натрия.

С июня по август 2018 г. в связи с выраженной активностью ЭОП (CAS=6 по шкале клинической активности EUGOGO) [11], экзофтальмом, ограничением подвижности глаз, снижением остроты зрения проведена пульс-терапия метилпреднизолоном в суммарной дозе 9500 мг с непродолжительным эффектом. По данным лабораторных анализов от 08.06.2018 г.: св. Т4 — 15,5 пмоль/л (9–20), св. Т3 — 3,53 пмоль/л (2,5–5,5), ТТГ — 0,215 мМЕ/л (0,25–3,5), антитела к рецептору ТТГ — 7,24 МЕ/л (0–1,75) на фоне приема 88 мкг левотироксина натрия. В сентябре 2018 г. бросила курить.

В октябре 2018 г. после отмены метилпреднизолона отмечена значительная отрицательная динамика. При проведении мультиспиральной компьютерной томографии (МСКТ) выявлен синдром вершины орбиты, или «апикального сгущения«, когда глазодвигательные мышцы сдавливают зрительный нерв (рис. 1, А, В). При обследовании в ФГБНУ «НИИ глазных болезней» поставлен диагноз: ЭОП крайне тяжелой степени, оптическая нейропатия с двух сторон, рестриктивное вертикальное косоглазие с горизонтальным компонентом. Бинокулярное двоение. Гипертропия слева. Эзотропия слева.

**Figure:**
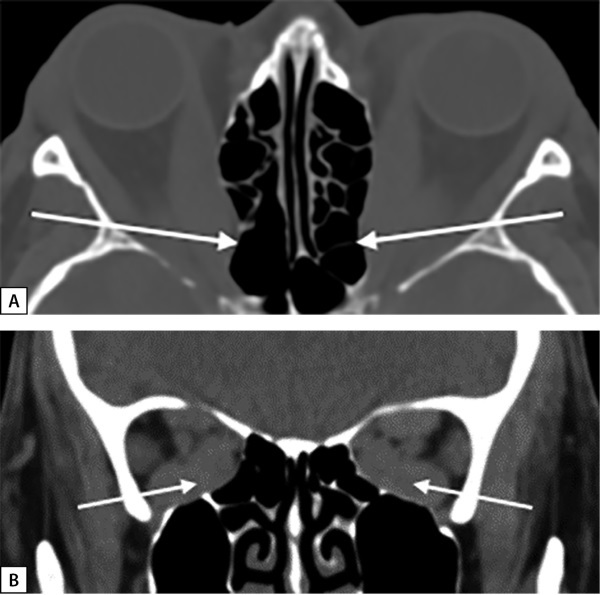
Рисунок 1. МСКТ орбит пациентки А.: А — увеличение медиальных прямых мышц. Деформация медиальной стенки в задней части, больше выраженная справа (аксиальная проекция), белые стрелки; В — апикальный синдром. Увеличение медиальных, нижних и верхних прямых мышц (корональная проекция), белые стрелки.

В связи с резистентностью к лечению ГК и двусторонней оптической нейропатией в ФГБНУ «НИИ глазных болезней» в октябре и ноябре 2018 г. были выполнены последовательно глубокая латеральная и медиальная (эндоназальная трансэтмоидальная) декомпрессия левой и правой орбиты. После операций достигнута четкая положительная динамика по зрительным функциям: повышение остроты зрения с 0,7 до 1,0; данным компьютерной периметрии и параметрам цветового зрения. В наиболее тяжелых случаях оптической нейропатии при больших мышцах эффективен максимальный объем декомпрессии — 2,5 стенок [12]. На МСКТ после операций на орбитах отмечается адекватное увеличение объема костной орбиты в задней части, необходимое при апикальном синдроме (рис. 2, А, В). В дальнейшем, последовательно до апреля 2019 г., выполнены: коррекция вертикально-горизонтального косоглазия и коррекция ретракции век с хорошим эффектом.

**Figure:**
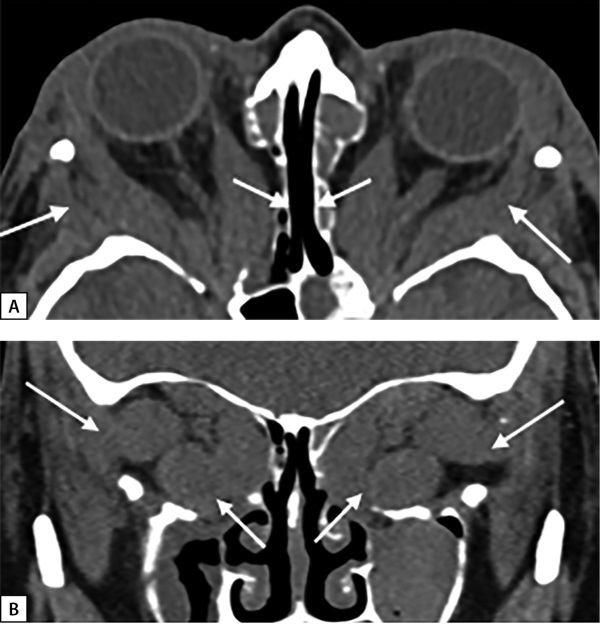
Рисунок 2. МСКТ орбит через 1 месяц после глубокой латеральной и медиальной декомпрессии: А — смещение увеличенных прямых мышц в сформированные костные окна (аксиальная проекция), белые стрелки; В — экспансия мягкотканных структур, прежде всего мышц за пределы резецированных наружной, медиальной и внутренней части нижней стенки орбиты (корональная проекция), белые стрелки.

В сентябре 2019 г. происходит увеличение аксиальной проминенции правого глаза на 2 мм и возобновление бинокулярного двоения. В связи с чем проведена пульс-терапия метилпреднизолоном (суммарно 1500 мг) с некоторой положительной динамикой. Признаков оптической нейропатии на этом сроке отмечено не было.

Зимой 2020 г. стала отмечать локальный отек и гиперемию голеней. Неоднократно обращалась к дерматовенерологу, эндокринологу, ревматологу по месту жительства. Верифицировать точный диагноз не удалось. Проводились различная терапия (десенсибилизирующие средства, ангиопротекторы, антибактериальные препараты) и местное лечение (топические ГК, смягчающие средства), без эффекта.

В октябре 2021 г. с диагнозом «первичный гипотиреоз в исходе субтотальной резекции ЩЖ по поводу БГ, медикаментозная компенсация, ЭОП тяжелой степени, неактивная фаза (CAS=1), вторичное косоглазие», пациентка была госпитализирована в ФГБУ «НМИЦ эндокринологии» Минздрава России. При осмотре в отделении: эутиреоидное состояние, незначительный отек век, острота зрения OD=1, OS=0,9, экзофтальмометрия OD=20, OS=17, ретракция верхнего века справа, движения глазных яблок ограничены вверх и влево. МСКТ орбит — картина экзофтальма, утолщения глазодвигательных мышц, жировой трансформации мышц. Пациентка осмотрена офтальмологом: ЭОП тяжелой степени, неактивная фаза, состояние после костной декомпрессии. Оперированное вторичное косоглазие. Артифакия. Невус радужки.

При осмотре нижних конечностей: кожа голеней и стоп уплотнена, в складку не собирается, при пальпации безболезненна, при надавливании ямки не остается. Цвет очага на левой голени розовый с четкими границами на передней поверхности и менее крупный очаг на задней поверхности бледно-розового цвета, на правой голени 2 очага на передней поверхности бледно-розового цвета с четкими границами.

В анализах: ТТГ 2,82 мМЕ/л (норма 0,25–3,5 мМЕ/л) на фоне приема 75 мкг левотироксина натрия, антитела к рецептору ТТГ 33,6 МЕ/л. При УЗИ ЩЖ выявлена остаточная ткань обеих долей, справа 10×8,5 мм, слева 8×11 мм.

При УЗИ мягких тканей нижних конечностей в средней трети правой голени по передней поверхности в зоне претибиальной дермопатии кожа толщиной 0,45–0,5 см, низкой эхогенности, граница с подкожно-жировой клетчаткой (ПЖК) размыта. Отмечается невыраженный отек ПЖК в медиальных участках поражения. В средней трети голени в ПЖК визуализируется жидкостное образование 0,3 см. В средней трети левой голени по задней и передней поверхностям кожа толщиной до 0,4 см, низкой эхогенности, граница с ПЖК размыта. В ПЖК отека нет. При проведении цветного допплеровского картирования (ЦДК) — васкуляризация не изменена. Заключение: эхографические признаки выраженного отека дермы в зонах поражения, единичной атеромы передней поверхности голени справа.

На основании клинической и ультразвуковой картины выставлен диагноз «претибиальная микседема». Проведено лечение: левотироксин натрия 75 мкг, преднизолон 300 мг на 300 мл физиологического раствора № 4, после инфузии преднизолона в/в инъекция фуросемида 20 мг. Параллельно проводилась FREMS-терапия с последующей компрессией нижних конечностей эластичным бинтом. Перед проведением получено добровольное согласие пациентки на данный вид лечения.

FREMS-терапия проводилась с помощью аппарата Aptiva (Lorenz Lifetech, Ozzano dell’Emilia [ранее Lorenz Biotech, Медолла], Италия), согласно протоколу лечения трофических ран голеней. Одноразовые электроды были наложены по периметру участков микседемы, установленный вольтаж составлял от 60 до 80 Гц на разных каналах, было задействовано 4 канала и 8 электродов от каждого канала (всего 32), методика наложения и точки расположения электродов представлены на рис. 3.

**Figure:**
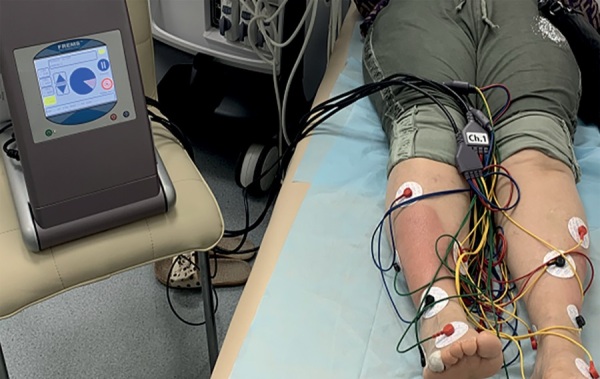
Рисунок 3. FREMS-терапия с помощью аппарата Aptiva (Lorenz Lifetech, Ozzano Dell’emilia). Представлена техника наложения электродов.

Лечение состояло из 5 сеансов длительностью 40 мин, 1 раз в 1–2 дня. После сеансов пациентке выполнялась эластическая компрессия вен нижних конечностей. На фоне лечения отмечена существенная положительная динамика в виде уменьшения отека, напряженности и интенсивности гиперемии очагов микседемы (рис. 4, А, В).

**Figure:**
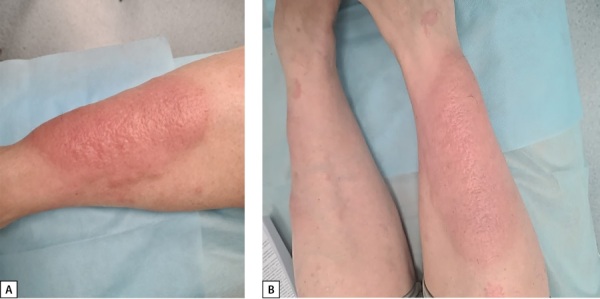
Рисунок 4. Фотография голени пациентки А.: А — фотография голени до проведения терапии; В — фотография голеней после проведения сеансов пульс-терапии и FREMS-терапии.

## ОБСУЖДЕНИЕ

Особенностью данного клинического случая является хронологическая последовательность развития БГ, ЭОП и претибиальной микседемы. Так, ЭОП развилась через 10 лет после манифестации диффузного токсического зоба и через 5 лет после субтотальной резекции ЩЖ. Претибиальная микседема развилась через 14 лет после манифестации БГ, через 9 лет после субтотальной резекции ЩЖ и через 4 года после развития ЭОП. Следует отметить, что у пациентки постоянно персистировали антитела к рецептору ТТГ (в последнем анализе — 33,6 МЕ/л) и визуализировалась остаточная ткань при УЗИ ЩЖ без тенденции к росту. Тяжелые проявления ЭОП с угрозой потери зрения, рефрактерность к лечению высокими дозами внутривенных ГК определили необходимость проведения костной декомпрессии орбит. Последним поражением явилось развитие претибиальной микседемы. Патогенез развития претибиальной микседемы не уточнен. Предполагается, что в развитии аутоиммунного процесса в тканях кожи претибиальной области участвуют иммунологические факторы, антитела к рецепторам ТТГ и инсулиноподобному фактору роста-1. Высокий уровень антител к рецептору ТТГ сохранялся у данной пациентки после субтотальной резекции ЩЖ в состоянии медикаментозного эутиреоза. В настоящее время эффективные методы лечения претибиальной микседемы отсутствуют. Ранее применявшиеся методы (местные ГК, пентоксифиллин, ритуксимаб, плазмаферез, внутривенное введение иммуноглобулина, октреотид, тепротумумаб) [13–17] имели незначительный и краткосрочный эффект. В нашем клиническом наблюдении использование комплексного подхода в тактике лечения кожных нарушений на основе применения пульс-терапии преднизолоном в сочетании с FREMS-терапией позволило добиться быстрого уменьшения выраженности симптомов претибиальной микседемы.

## ЗАКЛЮЧЕНИЕ

Учитывая достижение быстрого уменьшения клинических проявлений претибиальной микседемы, отсутствие побочных эффектов и хорошую переносимость, необходимо рассмотреть подобную тактику комбинированного применения пульс-терапии преднизолоном и FREMS-терапии для более углубленных исследований и большего числа наблюдений.

## ДОПОЛНИТЕЛЬНАЯ ИНФОРМАЦИЯ

Источники финансирования. Работа выполнена по инициативе авторов без привлечения финансирования.

Конфликт интересов. Авторы декларируют отсутствие явных и потенциальных конфликтов интересов, связанных с содержанием настоящей статьи.

Участие авторов. Перепелова М.А. — получение, анализ данных, написание статьи; Зайцева Е.Л. — проведение FREMS-терапии, написание статьи; Бессмертная Е.Г. — проведение офтальмологических исследований, одобрение финальной версии рукописи; Груша Я.О. — проведение хирургического лечения ЭОП, внесение в рукопись важной правки; Свириденко Н.Ю. — получение, анализ данных, внесение в рукопись важной правки; Галстян Г.Р. — получение, анализ данных, внесение в рукопись важной правки. Все авторы одобрили финальную версию статьи перед публикацией, выразили согласие нести ответственность за все аспекты работы, подразумевающую надлежащее изучение и решение вопросов, связанных с точностью или добросовестностью любой части работы.

Согласие пациента. Пациентка подписала добровольное информированное согласие на публикацию персональной медицинской информации в обезличенной форме в журнале «Проблемы эндокринологии».
